# Development of microsatellite markers for three at risk tiger beetles *Cicindela dorsalis dorsalis*, *C. d. media*, and *C. puritana*

**DOI:** 10.1186/s13104-020-04985-8

**Published:** 2020-03-23

**Authors:** Aaron W. Aunins, Michael S. Eackles, David C. Kazyak, Michael R. Drummond, Timothy L. King

**Affiliations:** 1 U.S. Geological Survey, Leetown Science Center, 11649 Leetown Road, Kearneysville, WV 25430 USA; 2 U.S. Fish and Wildlife Service, Virginia Field Office (Retired), 6669 Short Lane, Gloucester, VA 23061 USA

**Keywords:** Microsatellites, *Cicindela dorsalis dorsalis*, *Cicindela dorsalis media*, *Cicindela puritana*, Shotgun genomic sequencing

## Abstract

**Objective:**

Tiger beetles inhabiting sandy beaches and cliffs along the east coast of the United States are facing increasing habitat loss due to erosion, urbanization, and sea level rise. The northeastern beach tiger beetle *Cicindela dorsalis dorsalis* and Puritan tiger beetle *Cicindela puritana* are both listed as threatened under the Endangered Species Act of 1973, while the white beach tiger beetle *Cicindela dorsalis media* is not listed but has been declining. Extirpation of these beetles, in some cases from entire states, has isolated many populations reducing gene flow and elevating the risk for the loss of genetic variation. To facilitate investigations of population genetic structure, we developed suites of microsatellite loci for conservation genetic studies.

**Results:**

Shotgun genomic sequencing of all species identified thousands of candidate microsatellite loci, among which 17 loci were optimized and verified to cross-amplify within *C. d. media* and *C. d. dorsalis*, and eight separate loci were optimized for *C. puritana*. Most loci conformed to Hardy–Weinberg equilibrium, showed no evidence of linkage disequilibrium or null alleles, and revealed population genetic characteristics informative for natural resource managers among the populations tested.

## Introduction

Tiger beetles of the genus *Cicindela* are large diurnal predatory insects that tend to prefer sandy habitats near bodies of water such as river edges, and coastal beaches [1]. Many species along the North American Atlantic coast are declining due to the destruction of adult and larval beach habitat through increased development and recreational use, erosion, and sea level rise. The federally threatened northeastern beach tiger beetle *Cicindela dorsalis dorsalis*, which once was described as occurring in great swarms along beaches from Martha’s Vineyard, Massachusetts (MA) to New Jersey (NJ), and a common inhabitant of coastal beaches from MA south to Virginia (VA) is extirpated from much of its native range (United States Fish and Wildlife Service (USFWS) [2]). The white beach tiger beetle *C. d. media* native range overlaps with *C. d. dorsalis* and extends from NJ south to Florida (FL). However, while this species is also declining, it is generally considered more abundant than *C. d. dorsalis* [3]. The Puritan tiger beetle *C. puritana* is federally listed as threatened, and historically ranged from the Chesapeake Bay to Connecticut (CT), but is now reduced to a few isolated populations in Maryland (MD) and CT. While other tiger beetles co-occur with *C. d. media*, *C. d. dorsalis*, and *C. puritana*, these specific species are currently the focus of intense conservation efforts. To support their conservation, we developed a suite of microsatellite loci for population genetic research to facilitate estimation of the extent of gene flow, genetic diversity, and existence of metapopulations.

## Main text

### Methods

Multiple genomic shotgun DNA libraries of single individuals and pooled conspecifics were prepared from *C. d. media*, *C. d. dorsalis*, and *C. puritana* collected from throughout their native range. All samples were collected by the USFWS and provided to the U.S. Geological Survey (USGS) Leetown Science Center as whole beetles preserved in 95% ethanol. DNA was extracted from the head of each individual beetle using the DNEasy Blood and Tissue Kit (Qiagen, Germantown, MD). DNA was quantified using a Nanodrop spectrophotometer (ThermoFisher Scientific, Frederick, MD), and used for construction of libraries for Ion Torrent PGM sequencing. Sequence reads were generated from *C. puritana* (*n *= 1), *C. d. media* (*n *= 1), and *C. d. dorsalis* (*n *= 7) among 11 Ion Torrent sequencing chips. An additional library was sequenced on a 454 Junior for *n *= 1 *C. d. dorsalis*. All sequencing was performed at the USGS Leetown Science Center, Kearneysville, WV.

All sequence reads were imported into Qiagen CLC Genomics Workbench (ver 6.5.1). Quality and length trimming were performed with the following settings: ambiguous limit = 2, ambiguous trim = yes, quality limit = 0.015, minimum number of nucleotides in reads = 20, discard short reads = yes, remove 5′ or 3′ nucleotides = no. All quality trimmed *C. d. media* and *C. d. dorsalis* reads were concatenated into one file, and all quality trimmed *C. puritana* reads were concatenated into a separate file. We pooled the *C. d. media* and *C. d. dorsalis* samples since they are closely related subspecies, and microsatellite loci from one sub-species would have a high chance of success for cross-amplification in the other. Each fasta file was screened for di-, tri-, tetra-, penta-, and hexanucleotide microsatellite repeat motifs in the program QDD [4]. Settings for QDD included searching for a minimum of five repeats per motif, and a minimum sequence length of 80. The output of QDD included thousands of candidate microsatellite loci and primers designed using the integrated PRIMER 3 code [5]. From the two lists of candidate microsatellite loci, we chose to test primers for 30 loci in *C. d. media*/*C. d. dorsalis*, and 31 loci in *C. puritana*. Dinucleotide loci were avoided. Each sequence with a candidate microsatellite was blasted against the NCBI nt database, and none with any match to nt had strong similarity to organisms other than insects. Microsatellite loci were initially screened individually using M13 tailed primers [6]. Polymerase chain reactions were performed in 25 μl volumes, consisting of 10 ng of DNA, 1X PCR Buffer (Promega, Madison, WI), 0.25 μM of labeled forward primer, 0.5 μM of unlabeled reverse primer, 0.1 μM of labeled M13, 2.0 mM MgCl_2_, 0.2 mM of each dNTP, 0.25 units/μl Bovine Serum Albumin (New England Biolabs, Ipswich, MA), and 0.06 units/μl of Taq polymerase (Promega), using the following cycling conditions: 94 °C for 15 min, 29 cycles of 94 °C for 1 min, 58 °C for 45 s, and 72 °C for 45 s, 5 cycles of 94 °C for 1 min, 52 °C for 45 s, and 72 °C for 45 s, all followed by 72 °C for 10 min. PCR products for each locus were electrophoresed separately on an ABI 3130 Genetic Analyzer (ThermoFisher Scientific) automated DNA sequencer. Alleles were called using GeneMapper (ver. 4) (ThermoFisher Scientific) following the protocols described in King et al. [7].

The thirty *C. d. media* and *C. d. dorsalis* loci were initially tested on a sample of *n *= 8 *C. d. dorsalis* from Martha’s Vineyard, MA collected in 2013, and *n *= 8 from Cedar Island, MD collected in 2013. The thirty-one *C. puritana* loci were tested on *n *= 8 individuals collected from Little Cove Point, MD in 2013. Based on the amplification characteristics and levels of polymorphism within these test populations, 17 loci for *C. d. media*/*C. d. dorsalis* and eight loci for *C. puritana* were chosen for optimization in larger population samples (Tables 1 and 3). A multiplex PCR was designed for the *C. d. dorsalis/C. d. media* loci using the software Multiplex Manager [8], allowing the 17 loci to be run among four separate multiplex reactions (Table 1). Each multiplex PCR used the following concentration of reagents in a 15 µl reaction: 1.6X PCR Buffer (Promega, Madison, WI), 0.08 units/µl Taq polymerase (Promega), 0.2 µM of each forward and reverse primer, 0.3 mM dNTPs, and 3.75 mM MgCl_2_. Multiplex 1 and 3 utilized an annealing temperature of 56 °C, whereas 2 and 4 utilized 58 °C. Thermal cycling conditions were as follows: 94 °C for 2 min, 34 cycles of 94 °C for 30 s, 56/58 °C for 30 s, 72 °C for 90 s, followed by a final extension at 72 °C for 10 min. No multiplexed reactions were developed for the *C. puritana* microsatellite loci, which were genotyped using M13 tailed primers.

**Table 1:** Characteristics of 17 microsatellite loci in two collections of *Cicindela dorsalis dorsalis*, and one collection of *C. dorsalis media*

Locus	Primer sequences	Size range	Multiplex	Motif	Locus origin	Locus characteristic	MV *n *= 16 (*C. dorsalis dorsalis*)	CI *n *= 20 (*C. dorsalis dorsalis*)	FI *n *= 24 (*C. dorsalis media*)
Cdo4	F: ACAAAGAAAGAGACTCGCCC	141–156	4 FAM	AAC_(9)_	*Cdd*	*N*_A_	1.00	2.00	2.00
	R: CACACGTTTCAGGGATGGAC					*H*_O_	0.00	0.20	0.04
						u*H*_E_	0.00	0.19	0.04
						*A*_E_	1.00	1.22	1.04
						Microchecker null	No	No	No
						Micorochecker scoring error	No	No	No
						HWE *P*-value	NA	1.00	NA
Cdo5	F: TGTGTGTCCTATATTAGCTGATGC	139–148	3 VIC	AAT_(9)_	*Cdm*	*N*_A_	1.00	1.00	3.00
	R: GCGAGGCTATAAATATGCACTT					*H*_O_	0.00	0.00	0.54
						u*H*_E_	0.00	0.00	0.53
						*A*_E_	1.00	1.00	2.06
						Microchecker null	No	No	No
						Micorochecker scoring error	No	No	No
						HWE *P*-value	NA	NA	0.30
Cdo6	F: TCTCAGGATTACGAAGCAGAAA	123–129	1 VIC	AAT_(10)_	*Cdd*	*N*_A_	1.00	2.00	2.00
	R: GTACGATCGTCCTGCCCA					*H*_O_	0.00	0.50	0.17
						u*H*_E_	0.00	0.49	0.22
						*A*_E_	1.00	1.92	1.28
						Microchecker null	No	No	No
						Micorochecker scoring error	No	No	No
						HWE *P*-value	NA	1.00	0.30
Cdo7	F: CATTCTATATTCCTAAAGGGTTCC	105–111	4 VIC	AAT_(9)_	*Cdm*	*N*_A_	2.00	2.00	3.00
	R: CACCTACGACACACGTATAGTTACA					*H*_O_	0.13	0.05	0.21
						u*H*_E_	0.12	0.05	0.19
						*A*_E_	1.13	1.05	1.24
						Microchecker null	No	No	No
						Micorochecker scoring error	No	No	No
						HWE *P*-value	1.00	NA	1.00
Cdo8	F: AGCAGGCGTGTCGTGTTTAT	133–139	2 FAM	AAT_(9)_	*Cdm*	*N*_A_	3.00	2.00	3.00
	R: TGCTCAACCCTGAAGGAAGT					*H*_O_	0.88	0.30	0.46
						u*H*_E_	0.57	0.43	0.46
						*A*_E_	2.25	1.72	1.83
						Microchecker null	No	No	No
						Microchecker scoring error	No	No	No
						HWE *P*-value	0.01	0.28	1.00
Cdo11	CGTTTGGCAAGGTTAGTTC	123–135	2 PET	AAT_(9)_	*Cdm*	*N*_A_	2.00	2.00	5.00
	AAATTCCGTTTGACGGTGA					*H*_O_	0.31	0.35	0.71
						u*H*_E_	0.42	0.36	0.68
						*A*_E_	1.68	1.54	3.03
						Microchecker null	No	No	No
						Microchecker scoring error	No	No	No
						HWE *P*-value	0.53	1.00	0.99
Cdo13	TTCGATTCTTCGACTTGTTTCA	260–278	3 PET	AAC_(8)_	*Cdm*	*N*_A_	3.00	3.00	7.00
	TGAAATTTGATTGGCATACAGG					*H*_O_	0.63	0.45	0.92
						u*H*_E_	0.64	0.57	0.83
						*A*_E_	2.60	2.24	5.38
						Microchecker null	No	No	No
						Microchecker scoring error	No	No	No
						HWE *P*-value	0.60	0.50	0.44
Cdo15	GGGATAGAAAGGAGTTGGGTG	133–139	2 VIC	AAG_(8)_	*Cdm*	*N*_A_	1.00	1.00	3.00
	ACACTACTCGAGAACATCACCA					*H*_O_	0.00	0.00	0.21
						u*H*_E_	0.00	0.00	0.53
						*A*_E_	1.00	1.00	2.08
						Microchecker null	Yes	Yes	Yes
						Microchecker scoring error	Yes	Yes	Yes
						HWE *P*-value	NA	NA	0.00
Cdo21	AAGGCCGCAGTACAAGGAC	144–150	1 PET	AAT_(8)_	*Cdm*	*N*_A_	1.00	2.00	3.00
	AAACAGTTGTGCCGATAAATCTT					*H*_O_	0.00	0.15	0.58
						u*H*_E_	0.00	0.14	0.57
						*A*_E_	1.00	1.16	2.25
						Microchecker null	No	No	No
						Microchecker scoring error	No	No	No
						HWE *P*-value	NA	1.00	0.06
Cdo24	GAACAGGGACTGTTGTGGC	105–120	4 NED	AGC_(8)_	*Cdd*	*N*_A_	2.00	2.00	5.00
	ACCTGGTGGAGCGTCGTT					*H*_O_	0.06	0.30	0.48
						u*H*_E_	0.06	0.26	0.49
						*A*_E_	1.06	1.34	1.91
						Microchecker null	No	No	No
						Microchecker scoring error	No	No	No
						HWE *P*-value	NA	1.00	1.00
Cdo25	CGTTTATTGAGCCGGTGTTA	104–125	4 PET	CCG_(8)_	*Cdd*	*N*_A_	4.00	4.00	6.00
	GAACGGGCGATGTTTGAC					*H*_O_	0.31	0.65	0.92
						u*H*_E_	0.29	0.62	0.75
						*A*_E_	1.38	2.52	3.77
						Microchecker null	No	No	No
						Microchecker scoring error	No	No	No
						HWE *P*-value	1.00	0.59	0.12
Cdo28	AGGATGGTTATCAATTTGGC	228–243	1 FAM	AAT_(14)_	*Cdd*	*N*_A_	2.00	1.00	3.00
	CGACTAACAAATAGCCATACACA					*H*_O_	0.13	0.00	0.09
						u*H*_E_	0.23	0.00	0.24
						*A*_E_	1.28	1.00	1.31
						Microchecker null	Yes	Yes	Yes
						Microchecker scoring error	No	No	No
						HWE *P*-value	0.19	NA	0.00
Cdo29	CGCTGCCGATAGTACAAAT	135–175	1 FAM	ACAGT_(12)_	*Cdm*	*N*_A_	2.00	2.00	7.00
	CCCATCCCTGCCTTATTCTAT					*H*_O_	0.13	0.10	0.17
						u*H*_E_	0.23	0.49	0.54
						*A*_E_	1.28	1.92	2.11
						Microchecker null	Yes	Yes	Yes
						Microchecker scoring error	No	No	No
						HWE *P*-value	0.19	0.00	0.00
Cdo30	AACTTTGACCAATTGTGTTGG	143–149	2 NED	AAT_(10)_	*Cdd*	*N*_A_	2.00	2.00	3.00
	AAGGAAATTATTATTTGTTCGCAA					*H*_O_	0.50	0.25	0.46
						u*H*_E_	0.39	0.22	0.44
						*A*_E_	1.60	1.28	1.76
						Microchecker null	No	No	No
						Microchecker scoring error	No	No	No
						HWE *P*-value	0.51	1.00	0.33
Cdo33	GGATTGTAATTGAATGTGATTTGTG	266–286	3 FAM	ACCT_(9)_	*Cdm*	*N*_A_	2.00	2.00	5.00
	ATGTTATCTTCCGACCGTGG					*H*_O_	0.38	0.10	0.79
						u*H*_E_	0.44	0.10	0.79
						*A*_E_	1.75	1.11	4.38
						Microchecker null	No	No	No
						Microchecker scoring error	No	No	No
						HWE *P*-value	0.59	1.00	0.28
Cdo38	ATTCCACACGACTCCCTGTC	128–143	3 NED	AGC_(9)_	*Cdd*	*N*_A_	1.00	2.00	5.00
	TGCGGTGTTGCACTATTGAT					*H*_O_	0.00	0.55	0.79
						u*H*_E_	0.00	0.51	0.65
						*A*_E_	1.00	2.00	2.78
						Microchecker null	No	No	No
						Microchecker scoring error	No	No	No
						HWE *P*-value	NA	1.00	0.04
Cdo41	AAAGTCCACCGTTAGCACC	97–106	1 NED	AGC_(8)_	*Cdm*	*N*_A_	2.00	4.00	3.00
	GATAACGGTGAGGTGAGTCCA					*H*_O_	0.00	0.15	0.29
						u*H*_E_	0.44	0.55	0.60
						*A*_E_	1.75	2.13	2.42
						Microchecker null	Yes	Yes	Yes
						Microchecker scoring error	No	No	No
						HWE *P*-value	0.00	0.00	0.00
						*N*_A_ (mean, SE)	1.88, 0.21	2.12, 0.21	4.00, 0.39
						*H*_O_ (mean, SE)	0.20, 0.06	0.24, 0.05	0.46, 0.07
						u*H*_E_ (mean, SE)	0.23, 0.05	0.29, 0.05	0.29, 0.05
						*A*_E_ (mean, SE)	1.40, 0.12	1.54, 0.12	2.39, 0.29
						HWE P-value (Fisher’s method)	0.0052	0.0000	0.0000

“Locus” refers to the name assigned to the microsatellite containing sequence. “Size-range” is the bp size of the alleles genotyped. “Motif” is the repeat motif and number of repeats (in parentheses) identified from the sequence read in QDD. “Multi-plex” refers to the assignment of each locus to one of 4 multiplex PCR reactions along with the fluorophore used. See “Main text” for PCR conditions. “Locus origin” denotes whether the locus was derived from within *Cicindela dorsalis media* (*Cdm*) or *C. d. dorsalis* (*Cdd*) genomic sequence data, though all loci amplify in both subspecies. “Microchecker null” and “Microchecker scoring error” denote the results of tests in Microchecker for null alleles or scoring errors at each locus. The number of alleles (*N*_A_), effective number of alleles (*A*_E_), unbiased expected heterozygosity (u*H*_E_), observed heterozygosity (*H*_O_), were output by the Genalex software. The Hardy–Weinberg *P*-value is the *P*-value reported for each locus, and across all loci from Genepop using Fisher’s method at the bottom of the table. “MV” and “CI” refer to the Martha’s Vineyard and Cedar Island collections of *C. d. dorsalis*, and “FI” to the collection of *C. d. media* from Fisherman’s Island. See “Methods” of the text for details of these collections

#### Data analyses

Final testing of the microsatellite locus panel for the *C. d. media*/*C. d. dorsalis* loci was on population samples of *n *= 24 *C. d. media* from Fisherman’s Island, Virginia (FI; 37.086 N, − 75.947 W), *n *= 20 *C. d. dorsalis* from Cedar Island, Maryland (CI 37.937 N, − 75.892 W), and *n *= 20 *C d. dorsalis* from Martha’s Vineyard, MA (MV; 41.3498 N, − 70.464 W). For *C. puritana*, a population of *n *= 20 from Connecticut River, CT (location withheld), and *n *= 20 from Little Cove Point, MD (38.38635 N, − 76.385 W) were sequenced. All genotype data were analyzed in MICRO-CHECKER (ver 2.2.3) to assess the occurrence of null alleles, large allele dropout, and scoring errors [9]. Exact tests in GENEPOP [10] were used to determine if the distribution of genotypes at each locus conformed to Hardy–Weinberg equilibrium (HWE). Multi-locus tests of conformance to HWE were completed using Fisher’s method in Genepop. Linkage disequilibrium (LD) was tested for all pairs of loci using contingency tables in GENEPOP. All tests of HWE and LD tests in GENEPOP used the default Markov chain parameters. Significance levels for HWE and LD tests were adjusted using the sequential Bonferroni correction. To assess genetic diversity, observed and unbiased expected heterozygosity and the effective number of alleles were calculated in Genalex ver 6.5 [11, 12]. Finally, to evaluate the extent of genetic differentiation among populations, we calculated pairwise $F_{\text{ST}}^{{\prime }}$ in Genalex.

### Results and discussion

Raw sequencing reads from all specimens are deposited in the NCBI short read archive as BioSamples under the NCBI BioProject PRJNA563672 for *C. d. media* and *C. d. dorsalis*, and BioProject PRJNA563686 for *C. puritana*. Among the 9,703,887 quality trimmed *C. puritana* reads processed by QDD, 238,322 contained putative microsatellites. Similarly, among the 5,569,580 quality trimmed *C. d. media*/*C. d. dorsalis* reads, 66,576 were identified by QDD as containing putative microsatellites.

Summary statistics of the genotypes collected from 17 multiplexed loci tested in three population samples of *C. d. dorsalis* and *C. d. media* are presented in Table 1. There were no missing data. Microchecker identified locus Cdo15 as having potential scoring errors in addition to possible null alleles, while a few other loci were flagged as possibly having null alleles. There was no evidence of linkage disequilibrium among locus pairs within or among collections. Several loci were monomorphic in one of the *C. dorsalis dorsalis* collections, precluding tests of HWE in Genepop for these loci. All populations were out of HWE based on Fisher’s method examining *P*-values across all loci. The most polymorphic locus was locus Cdo13 with seven alleles in *C. d. media*, and the number of alleles averaged across loci was higher in *C. d. media* at four versus approximately two in the *C. d. dorsalis* collections. The expected heterozygosity averaged across loci was low and similar across the three collections ranging from 0.20–0.29, and effective number of alleles was small reflecting the low levels of heterozygosity. Pair-wise estimates of genetic differentiation ($F_{\text{ST}}^{{\prime }}$) were high and statistically significant among all collections ranging from 0.334 to 0.767 (Table 2). This suggests a high level of genetic differentiation, and suitability of these loci for characterizing population structure.

**Table 2:** Matrix of pair-wise $F_{\text{ST}}^{{\prime }}$ values (below diagonal) and *P*-values (above diagonal) between a collection of *Cicindela dorsalis media*, and two collections of *C. d. dorsalis*

	MV	CI	FI
MV	0.000	0.001	0.001
CI	0.563	0.000	0.001
FI	0.336	0.197	0.000

Pair-wise $F_{\text{ST}}^{{\prime }}$ was calculated in the Genalex ver 6.5 software, and significance was assessed using 999 permutations. “MV” and “CI” refer to the Martha’s Vineyard and Cedar Island collections of *C. dorsalis dorsalis*, and “FI” to the collection of *C. dorsalis media* from Fisherman’s Island. See the Methods section of the text for details of these collections

Complete genotypes were also obtained for the eight loci screened in two population samples of *C. puritana* (Table 3). Some loci were identified as having null alleles by Microchecker, but no loci were flagged as having scoring errors. All loci were polymorphic in at least one population. The Little Cove Point collection was out of HWE, while Connecticut River was in HWE based on Fisher’s method examining all loci. Like for the *C. d. dorsalis* and *C. d. media* loci, some of the *C. puritana* loci were not sufficiently polymorphic for HWE testing in Genepop. There was no evidence of linkage disequilibrium among locus pairs or among collections. The most polymorphic locus was CpuQ2 with six alleles in the LCP collection. While the average number of alleles was similar across populations, the number of alleles at each locus was variable between populations with no consistent pattern. Both observed and expected heterozygosity, as well as the effective number of alleles were similar and low in the two populations. Pair-wise $F_{\text{ST}}^{{\prime }}$ was large at 0.789 (*P* < 0.001) between the two *C. puritana* populations.

**Table 3:** Characteristics of eight microsatellite loci in two collections of *Cicindela puritana*

Locus	Primer sequences	Size range	Motif	Locus characteristic	CR *n *= 24	LCP *n *= 20
CpuQ1	F: GCGACTTATATACAGTTAGTGGTGT	218–251	AAT_(13)_	*N*_A_	1.00	5.00
	R: TGTCTAACAATTCTCTCGGATTGC			*H*_O_	0.00	0.65
				u*H*_E_	0.00	0.71
				*A*_E_	1.00	3.23
				Microchecker null	No	No
				Micorochecker scoring error	No	No
				HWE *P*-value	NA	0.6889
CpuQ2	F: ATAACGGGACACTGTGGACT	135–183	AAT_(12)_	*N*_A_	4.00	6.00
	R: ACACTTTGGCATTCAATTCGGA			*H*_O_	0.50	0.30
				u*H*_E_	0.66	0.74
				*A*_E_	2.81	3.57
				Microchecker null	Yes	Yes
				Micorochecker scoring error	No	No
				HWE *P*-value	0.1688	0.0000
CpuQ3	F: CTTCGTACGTCATGAAAGTACTTAT	196–214	ACT_(12)_	*N*_A_	3.00	4.00
	R: AACTTCAAGCTTTCTGGATCAGA			*H*_O_	0.60	0.40
				u*H*_E_	0.50	0.38
				*A*_E_	1.97	1.58
				Microchecker null	No	No
				Microchecker scoring error	No	No
				HWE P-value	1.0000	0.4148
CpuQ10	F: AAATTACGCGCGTGTACTGC	124–136	ATC_(11)_	*N*_A_	2.00	4.00
	R: AAGGGCTGATTCACGACACC			*H*_O_	0.05	0.50
				u*H*_E_	0.05	0.56
				*A*_E_	1.05	2.19
				Microchecker null	No	No
				Microchecker scoring error	No	No
				HWE P-value	NA	0.7256
CpuQ13	F: AGTTTCGCCACAAATCCTGC	116–140	AAT_(10)_	*N*_A_	5.00	3.00
	R: GGTAGGACCACCGCAGAATC			*H*_O_	0.75	0.25
				u*H*_E_	0.68	0.66
				*A*_E_	2.99	2.83
				Microchecker null	Yes	Yes
				Microchecker scoring error	No	No
				HWE P-value	1.0000	0.0006
CpuQ19	F: AGCAGCCACCTCTCTACACA	156–168	ACAT_(9)_	*N*_A_	3.00	3.00
	R: AGAGATATGTAGCCGGAAAGTAGC			*H*_O_	0.20	0.15
				u*H*_E_	0.41	0.44
				*A*_E_	1.65	1.75
				Microchecker null	Yes	Yes
				Microchecker scoring error	No	No
				HWE P-value	0.0053	0.0008
CpuQ23	F: TGATATGTGTTGACTTGGTGTAATG	146–162	ACTAT_(8)_	*N*_A_	3.00	2.00
	R: ACCATAATGCAACTTTATACATATGCT			*H*_O_	0.60	0.45
				u*H*_E_	0.65	0.50
				*A*_E_	2.75	1.96
				Microchecker null	No	No
				Microchecker scoring error	No	No
				HWE P-value	0.2368	0.6748
Cpu31	F: ATGATCTCCCGGTCTGTCCT	152–192	AAAT_(7)_	*N*_A_	3.00	2.00
	R: AATGTTCATTGATGTACTCGATCT			*H*_O_	0.35	0.05
				u*H*_E_	0.30	0.05
				*A*_E_	1.42	1.05
				Microchecker null	No	No
				Microchecker scoring error	No	No
				HWE P-value	1.0000	0.0000
				*N*_A_ (mean, SE)	3.00, 0.42	3.63, 0.50
				*H*_O_ (mean, SE)	0.38, 0.10	0.34, 0.07
				u*H*_E_ (mean, SE)	0.41, 0.10	0.50, 0.08
				*A*_E_ (mean, SE)	1.95, 0.28	2.27, 0.31
				HWE P-value (Fisher’s method)	0.1523	0.0000

“Locus” refers to the name assigned to the microsatellite containing sequence. “Size-range” is the bp size of the alleles genotyped. “Motif” is the repeat motif and number of repeats (in parentheses) identified from the sequence read in QDD. “Microchecker null” and “Microchecker scoring error” denote the results of tests in Microchecker for null alleles or scoring errors at each locus. The number of alleles (*N*_A_), effective number of alleles (*A*_E_), unbiased expected heterozygosity (u*H*_E_), observed heterozygosity (*H*_O_), were output by the Genalex software. The Hardy–Weinberg *P*-value is the *P*-value reported for each locus, and across all loci from Genepop using Fisher’s method at the bottom of the table. “CR” and “LCP” refer to the Connecticut River and Little Cove Point collections of *Cicindela puritana*. See “Methods” section of the text for details of these collections

Overall, the results of the initial application of these loci to a small set of samples herein suggest that they will have utility for assessing population structure and patterns of gene flow in other populations of *Cicindela* tiger beetles. In addition, the shotgun genomic sequencing approach we employed identified thousands of candidate loci, allowing for the development of additional markers if needed.

## Limitations

The number of populations and individuals examined so far is modest. Therefore, application of these microsatellite markers to additional populations of *C. d. media*, *C. d. dorsalis*, and *C. puritana* will reveal whether the levels of variation seen, such as a relatively small number of alleles per locus and low levels of heterozygosity, are typical among populations within these taxa. For a locus like Cdo15 in *C. d. media* and *C. d. dorsalis* identified by MICROCHECKER as having potential scoring errors, genotyping of more populations will help resolve whether this is truly a likely scoring error, or artifact of small sample size. Also, some individual loci strongly deviated from HWE and in most cases this was due to a heterozygote deficiency, most likely suggesting the occurrence of null alleles, though multiple processes such as non-random sampling can contribute to single locus departures from HWE [13]. Genotyping of additional populations with a higher sample size of individuals will help identify loci with consistent patterns of departure from HWE, the causes of which can be investigated further.

## Data Availability

The sequence reads from which microsatellites were identified are available through the NCBI BioProject database (https://www.ncbi.nlm.nih.gov/bioproject/) as bioproject PRJNA563686 for *C. puritana*, and PRJNA563672 for *C. d. media* and *C. d. dorsalis*. Microsatellite genotype data are available through USGS ScienceBase (10.5066/P9V9J5QZ)

## Abbreviations

HWE: Hardy Weinberg equilibrium
LD: Linkage disequilibrium
USGS: U.S. Geological Survey
USFWS: U.S. Fish and Wildlife Service
